# Extravasation into brain and subsequent spread beyond the ischemic core of a magnetic resonance contrast agent following a step-down infusion protocol in acute cerebral ischemia

**DOI:** 10.1186/2045-8118-11-21

**Published:** 2014-09-23

**Authors:** Tavarekere N Nagaraja, Kelly A Keenan, Madhava P Aryal, James R Ewing, Saarang Gopinath, Varun S Nadig, Sukruth Shashikumar, Robert A Knight

**Affiliations:** 1 Department of Anesthesiology, Henry Ford Hospital, 2799 West Grand Blvd, Detroit, MI 48202-2689, USA; 2 Department of Neurology, Henry Ford Hospital, Detroit, MI, USA; 3 Department of Physics, Oakland University, Rochester, MI, USA; 4 Department of Neurology, Wayne State University, Detroit, MI, USA; 5 Present address: Department of Radiation Oncology, University of Michigan, Ann Arbor, MI, USA

**Keywords:** Blood–brain barrier, Brain drug delivery, Penumbra, Perfusion-diffusion mismatch, Stroke

## Abstract

**Background:**

Limiting expansion of the ischemic core lesion by reinstating blood flow and protecting the penumbral cells is a priority in acute stroke treatment. However, at present, methods are not available for effective drug delivery to the ischemic penumbra. To address these issues this study compared the extravasation and subsequent interstitial spread of a magnetic resonance contrast agent (MRCA) beyond the ischemic core into the surrounding brain in a rat model of ischemia-reperfusion for bolus injection and step-down infusion (SDI) protocols.

**Methods:**

Male Wistar rats underwent middle cerebral artery (MCA) occlusion for 3 h followed by reperfusion. Perfusion-diffusion mismatched regions indicating the extent of spread were identified by measuring cerebral blood flow (CBF) deficits by arterial spin-labeled magnetic resonance imaging and the extent of the ischemic core by mapping the apparent diffusion coefficient (ADC) of water with diffusion-weighted imaging. Vascular injury was assessed via MRCA, gadolinium-diethylenetriaminepentaacetic acid (Gd-DTPA) penetration, by Look-Locker T_1_-weighted MR imaging after either a bolus injection (n = 8) or SDI (n = 6). Spatial and temporal expansion of the MRCA front during a 25 min imaging period was measured from images obtained at 2.5 min intervals.

**Results:**

The mean ADC lesion was 20 ± 7% of the hemispheric area whereas the CBF deficit area was 60 ± 16%, with the difference between the areas suggesting the possible presence of a penumbra. The bolus injection led to MRCA enhancement with an area that initially spread into the ischemic core and then diminished over time. The SDI produced a gradual increase in the area of MRCA enhancement that slowly enlarged to occupy the core, eventually expanded beyond it into the surrounding tissue and then plateaued. The integrated area from SDI extravasation was significantly larger than that for the bolus (*p* = 0.03). The total number of pixels covered by the SDI at its maximum was significantly larger than the pixels covered by bolus maximum (*p* = 0.05).

**Conclusions:**

These results demonstrate that the SDI protocol resulted in a spread of the MRCA beyond the ischemic core. Whether plasma-borne acute stroke therapeutics can be delivered to the ischemic penumbra in a similar way needs to be investigated.

## Background

Preserving the penumbra, the potentially salvageable area of brain tissue adjacent to an infarct core, is a primary objective following cerebral ischemia [1-3]. If successful, it can counter the expansion of the irreversibly damaged core and conserve penumbral cells for subsequent restorative and rehabilitative therapeutic interventions. Ways to achieve this are to reperfuse the ischemic tissue as quickly as possible and to deliver drugs that can enable the penumbral cells to withstand less than optimal conditions until a favorable milieu is re-established [4]. While approaches to initiate reperfusion including thrombolysis and mechanical clot retrieval are available, the delivery of therapeutics necessary for cell survival has proven difficult [5,6].

A major obstacle to brain drug delivery is the presence of a highly selective blood–brain barrier (BBB) [7]. Attempts have been made to overcome this obstacle by transiently opening the BBB via infusion of hyperosmotic mannitol [8], focused ultrasound [9,10] and ultrashort pulsed laser [11]. However, several brain pathologies including stroke, result in BBB opening [12-14] and evidence exists that this opening can be transient in acute stroke [15,16]. Post-stroke BBB opening results in extravasation of plasma-borne water and proteins into the brain [15,17] and their uptake by damaged or apoptotic cells in the vicinity [18,19]. Thus, it seems reasonable to assume that stroke-induced acute BBB opening, while being part of the pathology, may also be a potential conduit for the passage of therapeutics into the damaged brain. However, how to best use this route to preserve the penumbra has received relatively little attention.

Imaging modalities such as magnetic resonance imaging (MRI) or computer-aided tomography are frequently employed to measure tissue status in stroke and other cerebral pathologies. Of these, dynamic contrast-enhanced (DCE)-MRI provides the most data on brain vascular status. In acute stroke, the presence and magnitude of BBB opening needs to be evaluated as part of the patient screening process, since it can be a discriminating factor in deciding the course of thrombolytic treatment [20-22]. As the ischemic injury evolves, the BBB often becomes damaged allowing extravasation of water and plasma-borne substrates [23]. One potential method for assessing such post-stroke vascular injury is to use MRI to track the interaction of water molecules with a leaking magnetic resonance contrast agent (MRCA) [24]. In a typical study, an intravenous bolus injection of a Gd-based MRCA is given and quantitative T_1_-weighted images are acquired to visualize its enhancement. A bolus injection leads to a rapid rise in blood MRCA levels followed by an exponential fall. This continuously falling blood level may not be useful to study MRCA extravasation and its spread with extracellular fluid flow. MRCA backflux may also take place following a bolus injection if the BBB openings are relatively large [25]. Thus, despite its widespread use, a bolus injection may not be optimal to localize and quantify BBB damage [26,27]. In contrast, a step-down infusion (SDI) protocol can lead to near steady-state blood MRCA levels and generate higher signal-to-noise ratios [28]. It also results in superior visualization of BBB lesions and accurate localization of even very small BBB openings in acute stroke [29]. Therefore, we hypothesized that a SDI of an MRCA will be better than a bolus input for measuring the extent of vascular leakage in acute ischemia-reperfusion -induced BBB damage. A rat model of transient unilateral cerebral ischemia followed by reperfusion was used to test the hypothesis.

## Methods

### Animals and MRI

Experimental protocols were approved by the Institutional Animal Care and Use Committee. The MRCA, Gd-DTPA, was prepared at a concentration of 400 mM using published methods [30]. Male Wistar rats (Charles River, Wilmington, MA, USA) weighing ~300 g were anesthetized using 1.5% halothane in N_2_O:O_2,_ 70:30. Following published techniques [31], the right middle cerebral artery (MCA) was occluded using a 2 cm long, 4.0 nylon monofilament with a heat-blunted tip. A femoral artery and vein were cannulated with PE-50 tubing for monitoring arterial pressure and blood gasses, and for MRCA administration, respectively. The rat was placed on a feedback controlled, water-heated rubber mat in an acrylic holder equipped with a nose cone for inhalation anesthesia administration during MRI.

All studies were performed using a 7-Tesla, 20-cm bore superconducting Magnex magnet (Magnex Scientific Inc., Abingdon, UK) interfaced to a Bruker console (Bruker Biospin MRI, Inc., Billerica, MA, USA) and equipped with a 12-cm self-shielded gradient set capable of producing 25 G/cm gradients with 100µs rise times. Measurements of CBF, T_2_, T_1_ and diffusion-weighted imaging (DWI) were performed to localize the ischemic lesion following published methods [24,32-34]. At 3 h post-ictus, the holder was pulled out and the occluding nylon suture was withdrawn to initiate reperfusion and the rat returned to the magnet for additional imaging. Post-reperfusion images, identical to those acquired during occlusion, were obtained to assess the effects of reperfusion. At ~2.5 h post-reperfusion, baseline T_1_-weighted images (T_1_WI) were collected and then Gd-DTPA was intravenously administered either as a bolus or SDI [28]. The bolus injection consisted of 60µl of the Gd-DTPA stock diluted to 120µl with normal saline given through the femoral vein over about 5 s. For the SDI, the infusate was prepared by diluting 240µl of the Gd-DTPA stock solution to 4.0 ml with saline and approximately 3.5 ml was infused over a period of 5 min using a syringe pump following published methods [28]. Measurements of post-contrast enhancing areas were obtained every 2.5 minutes using a Look-Locker (LL) sequence [TR/TE = 2000 ms/2.2 ms, 5 slices, 2 mm thick, matrix 128 × 64, FOV = 32 mm] for approximately 25 minutes.

### Histology

After MR imaging, the animals were removed from the magnet and decapitated under deep anesthesia. The severed heads were instantly dropped into 2-methylbutane cooled to −45°C with dry ice. The brains were thus rapidly frozen *in situ* and carefully dissected from the heads in a chest freezer without thawing and embedded in Shandon M1 matrix (Thermo Electron Corporation, Pittsburgh, PA, USA). Coronal 20µm-thick frozen sections were taken at 400µm intervals spanning the brain from frontal to striate cortex. The sections were stained with cresyl violet (Nissl) and differences in staining intensity used to delineate the border between normal and ischemia-damaged tissue [34].

### Image analysis

Following off-line segmentation to exclude extra-cranial tissue, maps of ADC, CBF and subtraction LL-T_1_ images were made from data acquired during MCA occlusion and reperfusion [24,29,32-34]. A pulsed gradient spin-echo imaging sequence with progressively incremented diffusion-weighting (b-value) was used to measure ADC [34]. Maps of ADC were produced from a straight line least squares estimate of the slope from a plot of natural log of normalized image intensity vs. gradient b-value [34]. One standard deviation was subtracted from the mean contralateral ADC value to segment the ipsilateral ADC lesion and the number of pixels exhibiting low ADC values was counted. CBF rates (ml/min/100 g) were measured from a single central slice of CBF map generated from an arterial spin labeling sequence. The extent of T_2_ abnormality in the ipsilateral hemisphere was used to demarcate area of the CBF reduction and the number of such pixels was recorded. The pixel numbers of the ipsilateral and contralateral hemispheres were also measured separately. The difference in pixel numbers between the ipsi- and contralateral sides was considered to represent stroke-induced brain swelling, was subtracted from the ipsilateral value which was then used as the denominator to calculate hemispheric fractions of pixels with ADC and CBF lesions and contrast enhancement.

Following published methods [32], the MRCA-enhancing brain regions were selected from the LL-T_1_ maps by thresholding the pixel intensity of the post-contrast T_1_s to +2 standard deviations of the pre-MRCA image. The number of enhancing pixels in each rat at each time point was normalized to the largest area measured. The number of maximal enhancing pixels from each input as well as the area under the averaged curve for each input were measured and compared. All data were averaged for mean ± standard deviation (SD) for the two groups and significances inferred at *p* ≤ 0.05 using Studentâˆžs t-tests.

## Results

**Figure 1:**
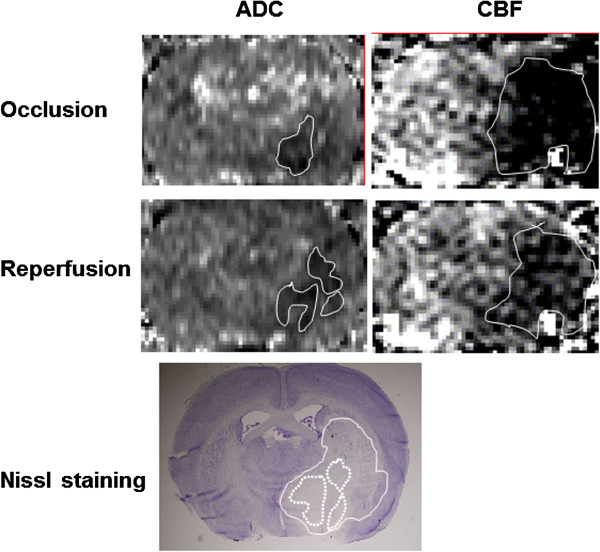
**Images to exemplify the areas of ADC and CBF deficits (demarcated by white outlines) during occlusion (top row), and after reperfusion (middle row).** Images were acquired 1.5-2.5 h post-stroke and 0.5-3.0 h post re-perfusion. Note the expansion of ADC lesion by a small extent and the partial restitution of CBF after reperfusion indicated by the appearance of relatively brighter pixels in the CBF deficit region. The animals were sacrificed following the MRCA enhanced imaging series (performed between 2.5 h and 3.0 h post-reperfusion) at approximately 3 h post-reperfusion and tissue sections were taken for histopathological assessment. The bottom photomicrograph shows the Nissl stained brain section from the same animal and demonstrates the stroke lesion (white outline) as seen on histopathology. It approximates the area of CBF deficit still persisting after reperfusion. An increase in Nissl staining intensity is faintly visible from medial to lateral part of the lesion, with the area of low intensity staining roughly matching the ADC lesion (regions marked by dotted lines).

**Figure 2:**
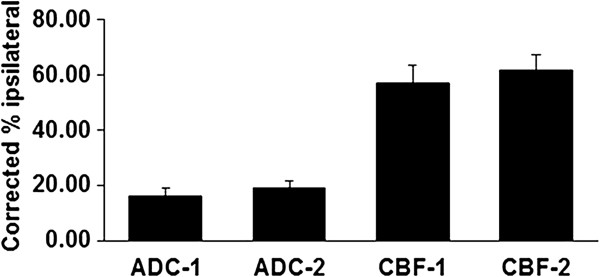
**Quantification of ADC and CBF lesion areas.** The pixels encompassing these lesions are expressed as a fraction of the ipsilateral hemisphere corrected for swelling. ADC-1, during MCA occlusion; ADC-2, after reperfusion; CBF-1, during MCA occlusion; CBF-2, after reperfusion. Images were acquired 1.5-2.5 h post-stroke and 0.5-3.0 h post re-perfusion.

Occlusion of the MCA reduced CBF to ~30% compared to the corresponding contralateral values in the region affected by occlusion. Reperfusion resulted in partial restoration of CBF (Figure 1). The extent of this rebound varied, with the preoptic area showing about 40% and the striatum about 50% of the corresponding contralateral flow. The ADC lesion size did not change significantly during the time from occlusion to reperfusion and was smaller than the corresponding CBF deficit area (Figure 2). Regional differences in BBB opening were also observed. Subcortical regions, such as the preoptic area and striatum, were affected more frequently (8 out of 12 rats) than neocortical regions. In the superior sagittal sinus, used to measure blood MRCA levels, the bolus injection resulted in a maximum ∆R_1_ (R_1_ = 1/T_1_, longitudinal relaxation rate and a measure of contrast agent concentration [24]) of 0.02 ms^−1^ in the first acquired image at 2.5 min corresponding to the peak blood level during that period and then decreased sharply over the next 2.5 min. The ∆R_1_ values continued to decline as the blood MRCA levels gradually fell and nearly leveled off 15–25 min. The SDI resulted in a similar initial increase (∆R_1_ = 0.02) over the first 2.5 min and then stayed at nearly the same level during the course of imaging. The relative areas under the curve (AUC) for the two arterial input functions were 1.5 ± 0.05 and 2.8 ± 0.5 in the bolus and SDI groups, respectively.

**Figure 3:**
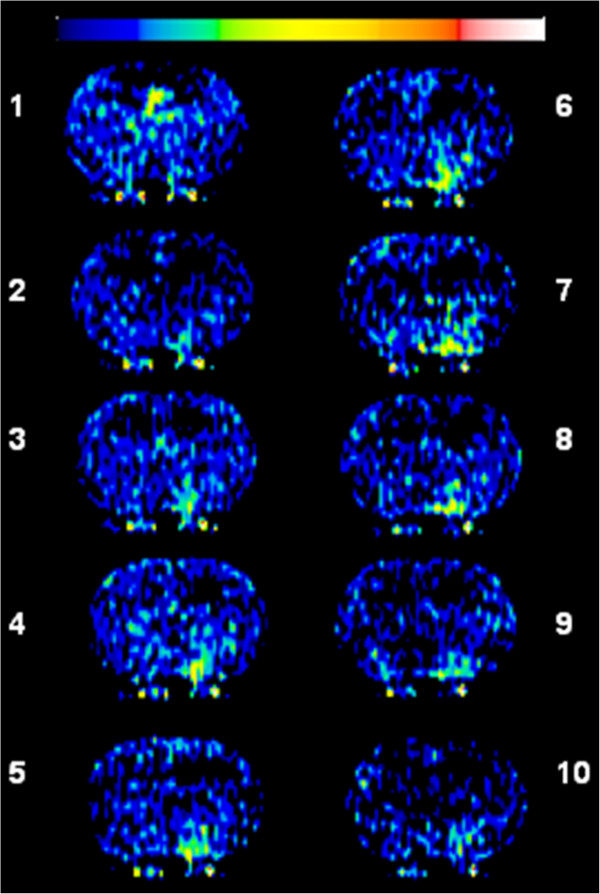
**Temporally acquired 10 post-contrast Look-Locker (L-L) T**_**1**_**-weighted images (T**_**1**_**WI) obtained over about 25 min following a bolus injection of Gd-DTPA.** The images demonstrate a region of enhancement that increased in size and signal intensity over the first 5 scans and then decreased over time. The color scale bar on top represents lowest to highest values in arbitrary units from left to right (black to white).

**Figure 4:**
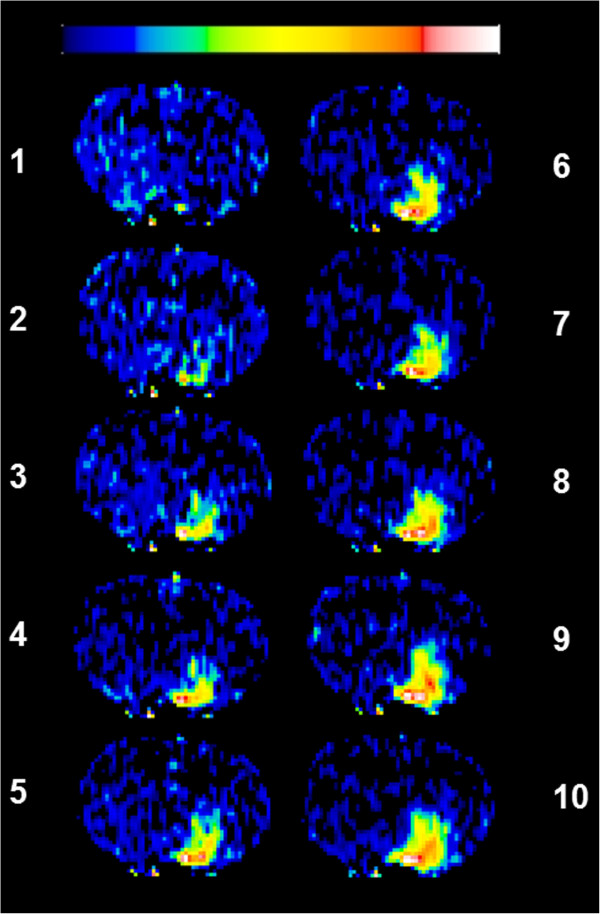
**Temporally acquired 10 post-contrast L-L T**_**1**_**WIs obtained over about 25 min following a step-down infusion of Gd-DTPA.** The images demonstrate a region of enhancement that increased in size and signal intensity over the first 4–5 scans before reaching a relatively steady state. The color scale bar on top represents lowest to highest values in arbitrary units from left to right (black to white). Note the persistent white- and red-tinged pixels suggestive of greater magnitude of leakage in the center of the enhancing region representing the probable source of leakage.

**Figure 5:**
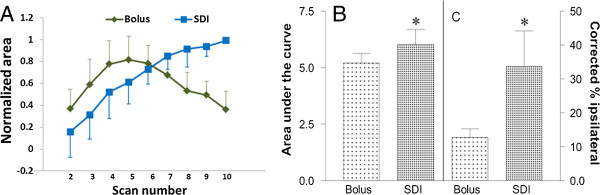
**Quantification of differences in spatial and temporal expansion of enhancing areas with bolus and SDI inputs. (A)** Normalized area measurements (mean ± standard deviation) are plotted as a function of time for the bolus (n = 8) and step-down infusion (n = 6) protocols. The first scan showed few enhancing pixels and is not plotted. Both inputs showed similarly increasing areas for the first 4 scans plotted. The enhancing area for the bolus input, however, began to decrease after the first 5 scans and continued to decrease for the remainder of the study (scans 6–10). Note that the mean last point has dropped down to nearly the same level as the first point shown (i.e., for the 2^nd^ scan). In comparison, the area of enhancement for the step-down protocol increased for the first 4–5 scans before reaching a relatively steady state (scans 6–10). **(B)** Plot showing the areas under the curve (AUC) for the two input functions which differed significantly for the bolus versus step-down injections on scans 8 and 9. The difference between the two mean values was significant (*p = 0.03). The values are scaled along the left Y-axis. **(C)** Plot showing the maximum enhancing pixels from the two inputs as corrected fractions of ipsilateral hemisphere. The SDI enhanced pixels covered about 30% whereas those from bolus covered about 12% of the total, the difference being statistically significant (*p = 0.05). These values are scaled along the right Y-axis; error bars represent standard error of the mean.

Representative images showing temporal leakage enhancement from the bolus and SDI inputs are shown in Figures 3 and 4, respectively. Initial enhancement after bolus injection was larger than after SDI, in line with the initial peak in blood levels immediately after injection (Figure 3). Conversely, the enhancement after SDI expanded comparatively slowly, but showed a steady and sustained enlargement (Figure 4). Quantification of the temporal expansion of contrast enhancement boundary from post-contrast images showed that for bolus injection the area of enhancement reached a peak during the first few scans after injection and then steadily decreased (Figure 5A). With a SDI input, however, the area of enhancement increased slowly in the beginning, but continued to increase throughout the imaging period and tending to plateau toward the end of the imaging duration (Figure 5A). The difference between the areas under the curves from the two input functions was significant (Figure 5B). The bolus at its maximum covered about 130 ± 70 pixels and the SDI, about 280 ± 200 pixels. The maximum hemispheric fraction area covered after the bolus injection enhancement (around the 5^th^ scan) was compared to that by SDI (mean of last two scans) and the difference was significant (Figure 5C).

## Discussion

These data support the hypothesis that following ischemia-reperfusion-induced BBB opening, an SDI input of Gd-DTPA aids in the expansion of extravasating MRCA front beyond the ischemic core. A comparison of pixel fractions for ADC and SDI (20% and 30%, respectively) corroborates this notion. The shape of the stepped input maintained a near-steady state plasma MRCA level and will have facilitated the positive driving force from blood-to-brain. Vasogenic edema is one of the direct consequences of BBB opening in acute stroke and results in influx of water into the brain. The Gd-based MRCA tracks the water protons and can accurately represent their position and movement. Contrast enhanced MRI, therefore, is a powerful tool to image and quantify these events.

One possible reason for the greater success of the SDI input could be the higher total amount of MRCA infused. It should be noted, however, that a similar quantity given as a bolus could have led to adverse cardiac reactions due to the very high osmolality (>1000 mOsm/kg) of the MRCA. Therefore, an SDI may also be the best way for physiologically delivering larger quantities of MRCA to demarcate small BBB openings [29] and, as seen by the present data, for tracking the fluid front. Reported blood-to-brain influx rates from bolus and SDI inputs were not statistically different, demonstrating that the SDI itself does not affect BBB permeability characteristics [28]. However, it would be useful to test the efficacy of a smaller quantity of MRCA delivered using an SDI input for providing a similar sustained enlarging enhancement pattern.

Two major mechanisms of fluid flow in brain extracellular space (ECS) are diffusion and bulk flow [35,36]. Diffusion by nature is expected to result in equivalent flow in all directions away from the source. Such unhindered flow is not possible in brain tissue that is characterized by a tortuous ECS with a normal volume fraction of ~20% [37-39] which may be further decreased in ischemia due to cell swelling. However, the temporal increase in contrast enhancing areas in the images suggests a still patent ECS at this time point after reperfusion. Bulk flow, since it occurs along paths of least resistance, is generally assumed to be along perivascular pathways [40]. Thus, a combination of diffusion and filtration in the ECS are likely the main mechanisms of the observed enlargement of the contrast enhancement. But, their relative contributions to the observed spread of extravasated Gd-DTPA are not clear from the present data. It is pertinent, however, that in cold-injury-induced vasogenic brain edema, linear distances traveled for tracers such as sucrose, fluorescein-labeled albumin and Evans blue-tagged albumin were larger than their theoretically predicted diffusion distances suggesting a sizeable contribution also by bulk flow under comparable circumstances [41]. In this context it is relevant to note that the Gd-DTPA used in the present study is a relatively small molecule (molecular weight, ~600 Da) and has no known uptake mechanisms in brain. As a result, it is likely to remain interstitial after leakage and spread over a larger area in the ECS. This assumed extravascular presence of Gd-DTPA is based on several previous observations as follows. The normal intravascular plasma distribution volume in brain is around 2% and the volume of extracellular space, about 20% as noted above. The Patlak plot which is employed by us to measure vascular kinetics in this model takes into account the intravascular levels while computing the brain distribution of Gd-DTPA. Such estimates for Gd-DTPA in stroke have routinely given values far exceeding the normal intravascular plasma distribution volume of 2% [23,24,32,34]. Moreover, vasodilation alone, even if present, cannot fully account for such large distribution volumes and that implies extravascular presence of the tracer.

Vasogenic edema in stroke is an important epiphenomenon, but has undergone little evaluation [14]. It is conventionally measured by post-mortem wet and dry tissue weight differences [42]. However, brain regional differences and directionality of edema fluid movement are lost by such measurements. Thus, *in vivo* measurements are essential to demonstrate any temporal fluctuations in acute BBB opening [43]. Apart from accurate demarcation of the expanding fluid front, there are other implications for this phenomenon such as the possibility of utilizing the edema fluid diffusion and convection to deliver therapeutics via BBB openings [44]. Thus, it is relevant that entry and uptake of blood-borne substrates into brain tissue after ischemia-reperfusion injury have been reported [15,18,19]. Also, the reported biphasic nature of such openings [16,43] may provide a direct, but transient, access to damaged brain under acute stroke.

Nevertheless, ischemia-induced changes in cerebral cytoarchitecture may impose further restrictions on effective brain drug delivery. It is known that diffusion of molecules after direct intracerebral injection is restricted [35,38]. Other brain drug delivery techniques have also met with limited success [7,45]. As noted above, brain has a tightly packed cell density, so most substances exhibit very low diffusion coefficients within the brain parenchyma due to either the tortuous ECS or molecular size [35]. In some disease conditions, including stroke, the ECS may be further constricted due to cell swelling. Therefore, it is not enough to just get drugs beyond the BBB, but also vital for them to traverse relatively long distances in effective concentrations. Accordingly, a timely combination of both BBB opening and an optimal drug administration schedule that augments the drug’s brain entry and its subsequent dispersion in brain seems warranted.

Previous data have shown that acute BBB opening in this model of stroke is size-selective and allows the extravasation of molecules up to several thousand Daltons in weight [19]. Of these, the smaller tracers are more likely to navigate longer distances than larger tracers [31]. Diffusion of a drug molecule may similarly be hindered owing to its size. Drug spread may also be restricted by neuronal and glial receptors and transporters that can result in cellular uptake after leakage and limit its passage. However, such an uptake that restricts the spread, but results in cellular protection would still be a positive outcome. A constant plasma drug level in such a scenario via an SDI input is likely to facilitate greater penetration into brain, if such drug delivery were to be attempted [44], and this study provides the proof of this concept.

## Conclusions

The window of opportunity to access the brain for several neuroprotective molecules is reported to be within 24 h after stroke onset [46]. It is conceivable that this phenomenon could be partly due to transient BBB opening. In addition, a sequential “rescue first, rehabilitate next” approach is reported to be beneficial to the ischemic brain [47]. Thus, taking advantage of acute BBB opening to deliver neuroprotective drugs could be important in such temporal therapy sequencing to treat stroke.

In this regard, we have shown that a step down infusion protocol provides greater penetration and warrants further investigations as a potential drug delivery tactic using BBB opening for acute stroke treatment.

## Abbreviations

MRCA: Magnetic resonance contrast agent; MCA: Middle cerebral artery; CBF: Cerebral blood flow; ADC: Apparent diffusion coefficient of water; Gd-DTPA: Gadolinium-diethylenetriaminepentaacetic acid; SDI: Step-down infusion; BBB: Blood–brain barrier; MRI: Magnetic resonance imaging; DCE: Dynamic contrast- enhanced; DWI: Diffusion weighted-imaging; T_1_WI: T_1_-weighted imaging; FOV: Field of view; LL: Look-locker; AUC: Area under the curve; ECS: Extracellular space; R_1_: 1/T_1_; SD: Standard deviation.

## Competing interests

The authors declare that they have no competing interests.

## Authors’ contributions

TNN prepared the contrast agent, performed the experiments, analyzed the data and drafted the manuscript; KAK and MPA analyzed the data; JRE wrote the MR imaging sequences and assisted in data acquisition; SG, VSN and SS assisted in MRI and histology data analysis; RAK performed the experiments, analyzed the data, edited the manuscript and funded the study. All authors have read and approved the final version of the manuscript.
